# Association of Diet Quality with Metabolic (Dysfunction) Associated Fatty Liver Disease in Veterans in Primary Care

**DOI:** 10.3390/nu15112598

**Published:** 2023-06-01

**Authors:** Natalia I. Heredia, Aaron P. Thrift, David J. Ramsey, Rohit Loomba, Hashem B. El-Serag

**Affiliations:** 1 Department of Health Promotion & Behavioral Sciences, School of Public Health, The University of Texas Health Science Center at Houston, Houston, TX 77030, USA; 2 Section of Epidemiology and Population Sciences, Department of Medicine, Baylor College of Medicine, Houston, TX 77030, USA; aaron.thrift@bcm.edu; 3 Dan L Duncan Comprehensive Cancer Center, Baylor College of Medicine, Houston, TX 77030, USA; 4 Houston VA HSR&D Center for Innovations in Quality, Effectiveness and Safety, Michael E. DeBakey Veterans Affairs Medical Center, Houston, TX 77021, USAhasheme@bcm.edu (H.B.E.-S.); 5 Section of Health Services Research, Department of Medicine, Baylor College of Medicine, Houston, TX 77030, USA; 6 Division of Epidemiology, Department of Family Medicine and Public Health, University of California at San Diego, San Diego, CA 92093, USA; roloomba@health.ucsd.edu; 7 NAFLD Research Center, Division of Gastroenterology, University of California at San Diego, La Jolla, CA 92093, USA; 8 Section of Gastroenterology and Hepatology, Department of Medicine, Baylor College of Medicine, Houston, TX 77030, USA

**Keywords:** metabolic (dysfunction) associated fatty liver disease, Veterans, Healthy Eating Index, Mediterranean Diet

## Abstract

Background: Diet is associated with metabolic (dysfunction)-associated fatty liver disease (MAFLD), but the dietary composition associated with MAFLD risk has not been well-examined. Aim: The purpose of this study was to assess the association of two healthy eating indices with the presence and severity of MAFLD in a sample of Veterans in a primary care setting. Methods: This was a single center cross-sectional study using a random stratified sample of Veterans enrolled in primary care. Participants underwent a Fibroscan and completed an interviewer-administered Diet History Questionnaire II from which we calculated the Healthy Eating Index-2015 and Alternate Mediterranean Diet Score. We used multivariable logistic regression models to assess associations of dietary quality with MAFLD. Results: We analyzed data from 187 participants, 53.5% of whom were female. On average, participants were 50.2 years of age (SD, 12.3 years) with an average BMI of 31.7 kg/m^2^. MAFLD was detected in 78 (42%) and at least moderate fibrosis in 12 (6%) participants. We found that the Alternate Mediterranean Diet Score was inversely associated with MAFLD (adjusted OR = 0.85, 95%CI 0.72–1.00), but controlling for BMI and total energy intake attenuated the association (adjusted OR = 0.92, 95%CI 0.74–1.15). We found no statistically significant associations between the Healthy Eating Index-2015 and MAFLD or advanced fibrosis. Discussion: We found that the Alternate Mediterranean Diet Score was significantly associated with lower MAFLD risk in Veterans; however, the association was mediated by BMI and total energy intake. A Mediterranean-style diet could potentially help reduce the risk of MAFLD, particularly if it helps control total energy intake and weight.

## 1. Introduction

Metabolic (dysfunction)-associated fatty liver disease (MAFLD) is a condition characterized by the build-up of fat in the liver, in the presence of overweight/obesity, type 2 diabetes, or at least two metabolic risk abnormalities that would indicate metabolic dysregulation (e.g., high waist circumference, elevated blood pressure, high triglycerides, low HDL, prediabetes) [1]. While the debate about which term to use is still ongoing, [2,3] recent studies indicate that there is high agreement between the MAFLD and non-alcoholic fatty liver disease (NAFLD) definitions, with this agreement potentially dropping as the prevalence of the underlying etiologies changes [4,5,6]. Regardless, the prevalence of both MAFLD and NAFLD is high in the United States population [4] and concerted efforts are needed to address disease pathogenesis and to inform primary and secondary prevention strategies. 

Treatment for MAFLD focuses on weight loss [7,8]. Dietary changes are closely linked with weight loss [9]. In the United States, the Dietary Guidelines for Americans 2015–2020 lay out the patterns of consumption recommended to adults, which include an eating pattern that consists of a variety of whole grains, fruits and vegetables, proteins, including lean meats, seafood and nuts, with limited added sugars, sodium, and refined grains [10]. The Healthy Eating Index-2015 assesses adherence to these guidelines and thus is an index of diet quality. Individuals can achieve a high score on the Healthy Eating Index-2015 by eating in a healthy United States-Style, Vegetarian, or Mediterranean-Style pattern, among other patterns [11]. The Mediterranean diet has been recommended as one potential dietary pattern that could reverse liver stiffness and prevent the progression to advanced hepatic disease [12,13]. The Mediterranean diet typically includes a variety of fruits, vegetables, legumes, cereals, and fish. The Alternate Mediterranean Diet Score is another diet quality index, [14] differing from the Healthy Eating Index-2015 in multiple ways such as the inclusion of alcohol and the exclusion of dairy, as well as the negative scoring of red and processed meat intake. While both the broader Healthy Eating Index-2015 and the more specific Alternate Mediterranean Diet Score have been shown to be associated with reduced risk of NAFLD in population-based cohort and cross-sectional studies in the general United States population [15,16,17,18], there are no studies that examined or compared the association of these two diet quality indices with MAFLD in a primary care-based United States population. 

Nearly half of the United States Veteran population receive their primary care through the Veterans Administration [19], and in general, this population tends to be older, sicker, and on more medication than the general United States public [20]. Obesity and type 2 diabetes are two of the main requirements of a MAFLD diagnosis, and there is a higher prevalence of obesity in Veterans compared with the general population [20,21,22], and Veterans have over twice the prevalence of type 2 diabetes compared with the general population [23]. Moreover, like the increase seen in the general population [24], there has been an increasing prevalence in NAFLD over time in Veterans [25] which, given the near perfect overlap between NAFLD/MAFLD cases in this population [6], means that MAFLD prevalence too has increased over time in this population. 

Given the burden in the United States Veteran population, the potentially protective aspects of high-quality diets, and the need to determine the association of the two healthy eating indices with MAFLD more specifically, the purpose of this study was to assess the association of the Healthy Eating Index-2015 and the Alternate Mediterranean Diet Score with the presence and severity of MAFLD in a sample of Veterans randomly selected from primary care practice. 

## 2. Methods

### 2.1. Design and Sample

This study was a single center cross-sectional study among primary care patients at the Michael E. DeBakey VA Medical Center in Houston, Texas, the parent study of which has been described elsewhere [6]. Briefly, the sampling frame included all Veterans 20–69 years of age and enrolled for primary medical care at the Michael E. DeBakey VA Medical Center in the VA Corporate Data Warehouse. We used a random stratified sampling strategy that included stratifying the Michael E. DeBakey VA Medical Center source population by sex and 10-year age-group (e.g., male 20–29, male 30–39, female 20–29), and performing random selection without replacement and with equal allocation from each stratum. We excluded Veterans with documented alcohol use disorder (defined as AUDIT-C scores ≥ 4 points for men and ≥3 for women) or active viral hepatitis. After screening 2437 randomly selected patient electronic medical records, we identified 1510 who fulfilled the study inclusion and exclusion criteria. These eligible patients were sent a letter inviting them to the study and asking for permission to contact them. Those who did not opt-out were then contacted by phone and invited to participate. Inclusion and exclusion criteria were verified at an in-person appointment and then consent was obtained. Prior to participation in the study, participants provided written informed consent, and thus Veterans that were unable to provide informed consent due to physical or mental incapacity were excluded from the study. In the present secondary data analysis, we only included participants with complete dietary and Fibroscan assessments. The study was conducted in accordance with both the Declarations of Helsinki and Istanbul and was approved by the Institutional Review Boards for the Michael E. DeBakey VA Medical Center and Baylor College of Medicine. 

### 2.2. Data Collection

Participants completed an interviewer-administered questionnaire and underwent a Fibroscan. Questionnaires collected information about age, sex, race/ethnicity, lifetime use of alcohol, smoking, personal medical history, and use of medications. Body mass index (BMI) was calculated from height and weight measurements taken at time of consent.

### 2.3. Measures

Diet Quality. We collected dietary data with a self-administered questionnaire, the Diet History Questionnaire II [26]. This food frequency questionnaire consists of over 153 items covering a multitude of foods/food groups as well as dietary supplements. The items ask about usual intake in the past 12 months.

We calculated the Healthy Eating Index-2015 using the Diet*Calc SAS macro appropriate to our questionnaire [27,28]. The Healthy Eating Index-2015 consists of 13 components, assessing both the adequate consumption of foods recommended by the Dietary Guidelines for Americans, including total vegetables, greens and beans, total fruits, whole fruits, dairy, whole grains, total protein foods, seafood and plant protein, as well as the minimal intake of certain foods, including added sugars, sodium, and refined grains. A ratio of monounsaturated and polyunsaturated fatty acids to saturated fatty acids was used to represent fatty acids. Points are assigned to each component, with a possible range of 0 to 100 for the overall Healthy Eating Index-2015 score, and higher scores indicating higher diet quality. 

From the Diet History Questionnaire II, we also calculated the Alternate Mediterranean Diet Score, constructed using population-specific cut points across a set of predefined dietary parameters [14]. Nine components define the Alternate Mediterranean Diet Score: fruits, vegetables (excluding potatoes), legumes, nuts, fish, whole grains, red and processed meats, alcohol intake, and a ratio of fat intake (monounsaturated to saturated fatty acids). We standardized all components for total energy (per 1000 kcal) [29]. We assigned individuals a value of 1 for each beneficial component (fruits, vegetables, legumes, nuts, fish, and whole grains) for which consumption was at or above the median and for each detrimental component (red and processed meat) for which consumption was at or below the median. In addition, we assigned a value of 1 for a fat intake ratio at or above the median. Alcohol intake was analyzed according to the National Institute on Alcohol Abuse and Alcoholism recommendations [30]. We defined moderate consumption as any consumption up to 13 drinks per week and assigned a score of 1. We assigned a score of zero for no alcohol consumption or consumption of more than 13 drinks per week. We computed the Alternate Mediterranean Diet Score as the sum of scores across the 9 components, with a range of 0 to 9 for the total score and a higher score indicating increased adherence to the Alternate Mediterranean Diet Score.

MAFLD. A Fibroscan (Echosens, Paris, France) was used to assess MAFLD. Fibroscan was used to obtain measurements of liver fat (controlled attenuation parameter) and fibrosis (liver stiffness measurement) using vibration-controlled transient elastography. Per standard protocol, the M probe was applied first, with the Fibroscan operator switching to the XL probe if needed based on the recommendations of the device. For each participant, the operator obtained a minimum of 10 measurements, and the median controlled attenuation parameter and liver stiffness measurement along with the interquartile range were calculated by the device. To ensure quality, all measurements were reviewed by a qualified hepatologist. We excluded examinations with an interquartile range/median ratio >0.30 when the median liver stiffness measurement is >7 kPa [31]. Reliable examinations included those with ≥10 measurements, a ratio of valid to total measurements of more than 60%, and interquartile range/median ratio of ≤30% [31,32,33]. We defined hepatic steatosis as a median controlled attenuation parameter of ≥290 dB/m [4,34]. We defined MAFLD cases as participants with hepatic steatosis and either BMI >25 kg/m^2^ or type 2 diabetes, or in the absence of either, two or more of the following: high triglycerides, hypertension, high LDL cholesterol, or low HDL cholesterol. We defined controls as participants not meeting the above conditions for MAFLD. In the subset of those with MAFLD, at least moderate hepatic fibrosis was defined as liver stiffness measurement >7 kPa [35].

### 2.4. Statistical Analyses

The Healthy Eating Index-2015 and Alternate Mediterranean Diet Score scores were stratified into tertiles based on their distribution in those without MAFLD. We used the Chi-Square test or Fisher’s exact test for categorical variables and the Student *t*-test or Mann–Whitney U-test for continuous variables to examine differences between participants with and without MAFLD. Factors with *p* < 0.20 in univariate models were considered for inclusion as covariates in the multivariable-adjusted models. We used univariate (Model 1) and multivariable-adjusted logistic regression models to calculate odds ratios (ORs) and associated 95% confidence intervals (95% CIs) for associations of dietary quality (Healthy Eating Index-2015 and Alternate Mediterranean Diet Score) for both MAFLD and moderate fibrosis. We adjusted for sex and age (Model 2) and subsequently also adjusted for total energy intake and BMI (Model 3). We did this separately for both diet quality indices, examining the continuous association of the indices, as well as by tertiles, for both outcomes. We also assessed an interaction term between both diet quality indices and sex on MAFLD. We used Stata 16 (StataCorp, College Station, TX, USA) for analyses and tests for statistical significance were two-sided at *p* < 0.05.

## 3. Results

A total of 187 participants were eligible for this analysis. On average, participants were 50.2 years of age (SD, 12.3 years) with an average BMI of 31.7 kg/m^2^ (Table 1). The study sample was almost 54% female. The sample was racially/ethnically diverse, with 39.5% non-Hispanic White, 36.4% non-Hispanic Black, and 18.2% Hispanic adults. Mean numbers of alcoholic drinks per day was 0.4 (SD, 1.0), and most of the sample reported drinking either never/rarely (42.2%) or less than 1 drink/day (50.8%). The average total energy intake was 2158 kcal/day. The mean Healthy Eating Index-2015 was 62.9 (SD, 9.6) from a range of 0 to 100. The mean Alternate Mediterranean Diet Score was 4.2 (SD, 2.0) from a range of 0 to 9. 

MAFLD was detected in 78 (42%) participants. MAFLD was associated with older age, sex (higher in men), and high BMI. While those without MAFLD had a higher diet quality (mean Healthy Eating Index-2015 = 63.1) compared with those with MAFLD (mean Healthy Eating Index-2015 = 61.9), the differences were not statistically significant between the two groups (*p* = 0.41). However, those without MAFLD reported a diet aligned with the Mediterranean-style (mean Alternate Mediterranean Diet Score= 4.5) significantly more frequently than those with MAFLD (mean Alternate Mediterranean Diet Score = 3.9; *p* = 0.04). Moderate fibrosis was detected in 12 (6.4%) participants. In the subgroup of individuals with MAFLD, those without moderate fibrosis had a higher diet quality (mean Healthy Eating Index-2015 = 62.3) and were more adherent to a Mediterranean-style diet (mean Alternate Mediterranean Diet Score = 4.0) than those with moderate fibrosis (mean Healthy Eating Index-2015 = 60.2 and mean Alternate Mediterranean Diet Score = 3.8), although the differences were not statistically significant. There were also no statistically significant demographic differences between MAFLD cases with and without moderate fibrosis.

We found no statistically significant associations between the Healthy Eating Index-2015 and MAFLD status in the univariate or any of the multivariable analysis (Table 2). Conversely, in univariate analyses, we found that those most adherent to a Mediterranean-style diet (i.e., those in Tertile 3), as measured by the Alternate Mediterranean Diet Score, had lower odds of MAFLD as compared with those least adherent to a Mediterranean-style diet (i.e., Tertile 1 of Alternate Mediterranean Diet Score; OR = 0.46, 95%CI 0.22–0.97). Similarly, in univariate analyses using a continuous measure of the Alternate Mediterranean Diet Score, we also found that a Mediterranean-style diet was inversely associated with MAFLD (OR = 0.85, 95%CI 0.73–0.99). This statistically significant association persisted when controlling for age and sex. However, the association was attenuated towards the null when we controlled for BMI and total energy intake (kcal) in the multivariable analysis (Model 3). There were no statistically significant interactions between the indices and sex on MAFLD. We found no statistically significant association between either diet quality index and moderate fibrosis (Table 3). 

## 4. Discussion

In this study, we assessed the association of diet quality with MAFLD risk in a sample of Veterans randomly selected from primary care practice. We examined two indices of diet quality, the Healthy Eating Index-2015, which assesses adherence to the Dietary Guidelines for Americans 2015–2020, a guideline which can be met through various eating patterns [11], and the Alternate Mediterranean Diet Score, which is a more specific index in that it assesses adherence to a Mediterranean-style diet [14].

We found that a higher Alternate Mediterranean Diet Score was significantly associated with reduced odds of having MAFLD, controlling for age and sex. The association was no longer significant when adjusting for BMI and total calorie intake. Several studies that assess the association of diet quality with NAFLD risk have found that the association is either mediated or confounded by BMI [36,37]. For example, a study testing this association in several European cohorts, albeit with different metrics to quantify adherence to the Mediterranean diet, also found that the association between this diet and NAFLD risk was attenuated when controlling for BMI [38]. Data from the Framingham Cohort indicated that change in the Alternate Mediterranean Diet Score over time was significantly associated with change in liver fat but attenuated after controlling for BMI [16]. Like in the findings in our study, this may mean that BMI partially mediates the association; in the Framingham Cohort, BMI mediated 38% of the association [16]. Other studies found the significant association persists when controlling for BMI [18,39]. On the other hand, using data from the Multiethnic Cohort Study, Park and colleagues found that the Alternate Mediterranean Diet Score was not associated with NAFLD risk even when just controlling for birth year, sex, race, and length of Medicare enrollment (similar to our Model 2) [15]. Some of these aforementioned studies ascertained NAFLD through less accurate means. Moreover, the samples were selected from different populations, which may have different underlying levels of severity of their condition. Lastly, these studies assessed the association of the eating indices with NAFLD, not MAFLD, which has different diagnostic criteria. However, even though a MAFLD diagnosis may be determined through elevated BMI, which is not true of NAFLD, the varying levels within what is “elevated” BMI (i.e., BMI of 25 kg/m^2^ which is overweight vs. a BMI of 35 kg/m^2^ which is Class II obesity) are very different metabolically, and thus, BMI may still explain much of the association between diet quality and MAFLD risk. There is a need for additional prospective studies with strong assessments to ascertain both diet and MAFLD to confirm if there is an association specifically between the Healthy Eating Index-2015 and/or Alternate Mediterranean Diet Score and MAFLD risk when controlling for BMI and/or total energy intake, as well as to investigate if BMI and/or total energy intake partially or fully mediates the association. 

We did not find statistically significant associations between the Healthy Eating Index-2015 and MAFLD. This finding contrasts with that of Park and colleagues, who found a statistically significant association between Healthy Eating Index-2015 and NAFLD across all models [15]. In other United States-based cohort studies, other metrics of overall diet quality, including a previous version of the Healthy Eating Index, have also shown an association with reduced risk of NAFLD [18,36,40]. However, all of these studies used different methods of ascertaining NAFLD, including abdominal Magnetic Resonance Imaging scans [40], non-contrast computed tomography [36], ultrasonography [18], and International Classification of Diseases codes [15].

The Healthy Eating Index-2015 is a measure of diet quality and adherence to the Dietary Guidelines for Americans 2015–2020 [11], which can be met in a variety of ways, including a Mediterranean-style diet [10], whereas the Alternate Mediterranean Diet Score is more specifically a metric of adherence to the Mediterranean-style diet [14]. Both indices include vegetables, fruits, whole grains, legumes, and fatty acids. However, the Alternate Mediterranean Diet Score excludes potatoes from the total vegetable calculation, while the Healthy Eating Index-2015 “rewards” whole fruit intake in addition to total fruit intake. While both indices include fatty acids, the Alternate Mediterranean Diet Score uses a ratio of monounsaturated to saturated fat, while the Healthy Eating Index-2015 uses a ratio of monounsaturated and polyunsaturated fatty acids to saturated fatty acids. The Healthy Eating Index-2015 also takes into consideration that the diet be low in refined grains, sodium, and added sugars—categories that are not present in the Alternate Mediterranean Diet Score—and excludes alcohol. While both indices are concerned with protein intake, especially in the form of fish, the Alternate Mediterranean Diet Score specifically looks at fish and red and processed meats, while the Healthy Eating Index-2015 more globally categorizes these foods as total protein foods and seafood and plant protein together. Lastly, the Alternate Mediterranean Diet Score excludes dairy altogether and additionally includes a nuts category. Taken together, this provides ample reasons for there to be differences in the association of the two indices with MAFLD. Given that a Mediterranean-style diet is what is specifically recommended for MAFLD [12], it is then unsurprising to find a statistically significant association with the Alternate Mediterranean Diet Score but not Healthy Eating Index-2015 when not considering total energy intake. However, given the impact of total energy intake on the models, it appears that total energy consumption continues to be important, despite the underlying pattern, as suggested by clinical practice guidelines [7]. Indeed, other studies have seen an interaction between diet quality and total energy intake on NAFLD [41] that should be further explored in MAFLD more specifically. 

This study has several limitations. First, this is a cross-sectional study, limiting the ability to infer causality. Second, we had a limited sample size, which prevented us examining the association of diet quality with MAFLD by fibrosis or cirrhosis status. The diet indices used in this study pulled data from a Food Frequency Questionnaire, the Diet History Questionnaire II, which has more systematic measurement error as compared with 24-h recalls [42,43,44]. However, this study is the first to investigate the association of diet quality with MAFLD. Moreover, our sample was relatively diverse and was randomly selected from primary care patients thus reducing potential selection bias related to disease severity and testing bias.

## 5. Conclusions

In this study of Veterans, we found that a higher Alternate Mediterranean Diet Score was significantly associated with lower MAFLD risk; this association was possibly mediated by BMI and total energy intake. A Mediterranean-style diet could potentially help reduce risk of MAFLD, particularly if it helps keep total energy intake and weight in check. 

## Figures and Tables

**Table 1:** Characteristics of the MAFLD cases and control in the VA cohort.

Variable M (SD) or n (%)	Full Sample N = 187	MAFLD			Moderate Fibrosis		
		Yes n = 78	No n = 109	*p*-Value	Yes n = 12	No n = 66	*p*-Value
Age in years, Mean (SD)	50.2 (12.3)	53.6 (11.2)	47.8 (12.6)	**0.001**	52.5 (10.7)	53.8 (11.4)	0.717
Sex							
Male	87 (46.5%)	43 (55.1%)	44 (40.4%)	**0.046**	8 (66.6%)	35 (53.0%)	0.532 ^§^
Female	100 (53.5%)	35 (44.9%)	65 (59.6%)		4 (33.3%)	31 (47.0%)	
Race/Ethnicity							
Non-Hispanic White	74 (39.5%)	35 (44.9%)	39 (35.8%)	0.418	2 (16.7%)	33 (50.0%)	0.057
Non-Hispanic Black	68 (36.4%)	23 (29.5%)	45 (41.3%)		7 (58.3%)	16 (24.2%)	
Hispanic	34 (18.2%)	15 (19.2%)	19 (17.4%)		3 (25.0%)	12 (18.2%)	
Other	11 (5.9%)	5 (6.4%)	6 (5.5%)		0 (0.0%)	5 (7.6%)	
BMI, kg/m^2^							
Mean (SD)	31.7 (6.5)	35.1 (6.0)	29.3 (5.8)	**<0.001**	37.8 (5.8)	34.6 (6.0)	0.093
<25 kg/m^2^	26 (13.9%)	0 (0.0%)	26 (23.9%)	**<0.001 ^§^**	0 (0.0%)	0 (0.0%)	0.109 ^§^
25–30 kg/m^2^	54 (28.9%)	15 (19.2%)	39 (35.7%)		0 (0.0%)	15 (22.7%)	
≥30 kg/m^2^	107 (57.2%)	63 (80.8%)	44 (40.4%)		12 (100%)	51 (77.3%)	
Alcohol use, average drinks/day							
Mean (SD)	0.4 (1.0)	0.3 (0.4)	0.5 (1.3)	0.155	0.2 (0.3)	0.3 (0.5)	0.568
Never/rarely	79 (42.2%)	37 (47.4%)	42 (38.6%)	0.644 ^§^	5 (41.7%)	32 (48.5%)	0.876 ^§^
<1 drink/day	95 (50.8)	37 (47.4%)	58 (53.2%)		7 (58.3%)	30 (45.4%)	
1–2 drinks/day	10 (5.4%)	3 (3.9%)	7 (6.4%)		0 (0.0%)	3 (4.6%)	
>2 drinks/day	3 (1.6%)	1 (1.3%)	2 (1.8%)		0 (0.0%)	1 (1.5%)	
Total energy, kcal/day	2158 (2120)	1822 (1003)	2399 (2624)	0.066	1715 (447)	1841 (1075)	0.692
Healthy Eating Index 2015							
Mean (SD)	62.6 (9.6)	61.9 (10.0)	63.1 (9.2)	0.410	60.2 (12.3)	62.3 (9.6)	0.512
Tertile 1	60 (32.1%)	24 (30.8%)	36 (33.0%)	0.714	5 (41.7%)	19 (28.8%)	0.494 ^§^
Tertile 2	68 (36.4%)	31 (39.7%)	37 (34.0%)		3 (25.0%)	28 (42.4%)	
Tertile 3	59 (31.5%)	23 (29.5%)	36 (33.0%)		4 (33.3%)	19 (28.8%)	
Alternate Mediterranean Diet Score							
Mean (SD)	4.2 (2.0)	3.9 (1.9)	4.5 (2.0)	**0.038**	3.8 (2.3)	4.0 (1.9)	0.721
Tertile 1	67 (35.8%)	33 (42.3%)	34 (31.2%)	0.119	7 (58.3%)	26 (39.4%)	0.306
Tertile 2	65 (34.8%)	28 (35.9%)	37 (33.9%)		2 (16.7%)	26 (39.4%)	
Tertile 3	55 (29.4%)	17 (21.8%)	38 (34.9%)		3 (25.0%)	14 (21.2%)	

Notes: ^§^ Fisher’s Exact Test. Bold indicated statistical significance at *p* < 0.05.

**Table 2:** Association between two diet quality indices (modeled separately) and MAFLD.

	Model 1 OR 95% CI	Model 2 OR 95% CI	Model 3 OR 95% CI
Healthy Eating Index 2015 (continuous)	0.99 (0.96–1.02)	0.99 (0.96–1.02)	0.99 (0.96–1.03)
Healthy Eating Index 2015			
Tertile 1	Ref	Ref	Ref
Tertile 2	1.26 (0.62–2.54)	1.38 (0.66–2.89)	1.66 (0.71–3.91)
Tertile 3	0.96 (0.46–2.00)	1.00 (0.46–2.20)	1.28 (0.50–3.27)
Alternate Mediterranean Diet Score (continuous)	0.85 (0.73–0.99) *	0.85 (0.72–1.00) *	0.92 (0.74–1.15)
Alternate Mediterranean Diet Score			
Tertile 1	Ref	Ref	Ref
Tertile 2	0.78 (0.39–1.55)	0.79 (0.38–1.62)	0.75 (0.32–1.80)
Tertile 3	0.46 (0.22–0.97) *	0.49 (0.23–1.07)	0.77 (0.27–2.20)

Notes: Model 1: Univariate, Model 2: adjusted for age and sex, Model 3: Model 2 + BMI and kcal. * *p* < 0.05.

**Table 3:** Association between two diet quality indices (modeled separately) and moderate fibrosis.

	Model 1 OR 95% CI	Model 2 OR 95% CI	Model 3 OR 95% CI
Healthy Eating Index-2015 (continuous)	0.98 (0.93–1.03)	0.99 (0.93–1.04)	0.99 (0.94–1.05)
Healthy Eating Index-2015			
Tertile 1	Ref	Ref	Ref
Tertile 2	060 (0.18–2.00)	0.61 (0.18–2.07)	0.70 (0.20–2.43)
Tertile 3	0.70 (0.21–2.35)	0.80 (0.23–2.77)	0.88 (0.24–3.28)
Alternate Mediterranean Diet Score (continuous)	0.86 (0.67–1.11)	0.88 (0.68–1.14)	0.93 (0.69–1.25)
Alternate Mediterranean Diet Score			
Tertile 1	Ref	Ref	Ref
Tertile 2	0.54 (0.17–1.70)	0.57 (0.18–1.82)	0.54 (0.16–1.84)
Tertile 3	0.37 (0.10–1.45)	0.41 (0.10–1.63)	0.48 (0.10–2.27)

Notes: Model 1: Univariate, Model 2: adjusted for age and sex, Model 3: Model 2 + BMI and kcal.

## Data Availability

The data is available upon reasonable request to the corresponding author.
